# Peptide B targets soluble guanylyl cyclase α1 and kills prostate cancer cells

**DOI:** 10.1371/journal.pone.0184088

**Published:** 2017-08-31

**Authors:** Jun Zhou, Shuai Gao, Chen-Lin Hsieh, Mamata Malla, Lirim Shemshedini

**Affiliations:** Department of Biological Sciences, University of Toledo, Toledo, Ohio, United States of America; National Health Research Institutes, TAIWAN

## Abstract

Among androgen-regulated genes, soluble guanylyl cyclase α1 (sGCα1) is significant in promoting the survival and growth of prostate cancer cells and does so independent of nitric oxide (NO) signaling. Peptides were designed targeting sGCα1 to block its pro-cancer functions and one peptide is discussed here. Peptide B-8R killed both androgen-dependent and androgen-independent prostate cancer cells that expressed sGCα1, but not cells that do not express this gene. Peptide B-8R induced apoptosis of prostate cancer cells. Importantly, Peptide B-8R does not affect nor its cytotoxicity depend on NO signaling, despite the fact that it associates with sGCα1, which dimerizes with sGCβ1 to form the sGC enzyme. Just as with a previously studied Peptide A-8R, Peptide B-8R induced elevated levels of reactive oxygen species (ROS) in prostate cancer cells, but using a ROS-sequestering agent showed that ROS was not responsible the cytotoxic activity of Peptide B-8R. Interestingly, Peptide B-8R induced elevated levels of p53 and phosphorylated p38, but neither of these changes is the cause of the peptide’s cytotoxicity. Additional drugs were used to alter levels of iron levels in cells and these studies showed that Peptide B-8R activity does not depend on Ferroptosis. Thus, future work will be directed at defining the mechanism of cytotoxic action of Peptide B-8R against prostate cancer cells.

## Introduction

Because of the importance of androgen and the Androgen Receptor (AR) in both development and progression of prostate cancer [1, 2], existing therapy for this disease focuses on androgen deprivation or anti-androgen therapy[3]. While the anti-androgen drugs like Casodex have been used for many years, these drugs are not effective against the lethal form of the disease, castration-resistant prostate cancer (CRPC) [4]. More recent findings have led to the development new drugs to treat CRPC, such as the “second generation” anti-androgen Enzalutamide [5] and Abiraterone that targets androgen synthesis [6]. However, these drugs have shown limited efficacy in treating CRPC, providing patients only a few months of added survival time [7]. This finding necessitates the identification of new targets that can lead to new therapies.

Since AR acts by regulating gene expression in prostate cancer [8], downstream target genes of AR have important functions in this disease and against which novel therapeutic can be directed. Indeed, many androgen-regulated genes have been identified and shown to be important in prostate cancer, including PSA (prostate specific antigen) [9], hKLK2 (human kallikrein-2) [10], TMPRSS2 (transmembrane protease, serine 2) [11], ETV1 (Ets variant gene 1) [12], and sGCα1 (soluble guanylyl cyclase α1) [13]. The sGCα1 gene is known classically to encode one of the two subunits that comprise sGC, the enzyme that mediates nitric oxide (NO) signaling [14]. Our published data have shown that sGCα1 is an AR target gene that is over-expressed in prostate tumors at both the mRNA and protein levels, while expression of sGCβ1, its dimerization partner to form the sGC enzyme [14], is not significantly elevated [13]. Further, over-expressed sGCα1 mediates the proliferation of prostate cancer cells in the absence of androgen, mimicking the cellular growth that is observed with androgen [13]; this proliferative effect of sGCα1 is independent of NO signaling [13]. One target of the sGCα1 protein is p53, with which it associates and mediates its cytoplasmic sequestration, thereby providing prostate cancer cells an increased ability to survive and grow [15]. Interestingly, sGCα1 and p53 are found in a larger protein complex in prostate cancer cells, which also contains the CSN4 and CSN5 subunits of the COP9 Signalosome (CSN) [16] and Casein Kinase 2 (CK2) [17]. This complex and the CK2 enzyme activity regulate the protein levels of sGCα1 positively and p53 negatively and in this way promote the proliferation of prostate cancer cells [16].

Based on our published data described above, sGCα1 is an important androgen-regulated gene for prostate cancer and thus makes a good novel therapeutic target. We have targeted sGCα1 using peptides that bind this protein. One of these peptides, A-8R, has strong cytotoxic and anti-tumor activities against prostate cancer cells [18]. We have recently studied a new peptide, B-8R, that also binds to sGCα1 and kills prostate cancer cells by apoptosis. While Peptide B-8R induced the generation of reactive oxygen species (ROS), as does Peptide A-8R [18], ROS is a consequence of rather than responsible for cell death, unlike Peptide A-8R [18]. Interestingly, Peptide B-8R also caused elevated p53 protein levels and activation of p38 (phosphorylation of p38), both of which can lead to apoptosis of prostate cancer cells. However, the elevated levels of p53 and phosphorylated p38 are not responsible for Peptide B-8R cytotoxicity and mechanism remains unidentified.

## Materials and methods

### Cell culture and androgen treatment

LNCaP, C81, CWR-22Rv1, PC-3 and COS7 cells were grown as previously [13]. MR49F cells were cultured in RMPI-1640 medium with 10% FBS and 10 μM Enzalutamide. For androgen (R1881) treatment, LNCaP cells were grown in medium containing 2% FBS extracted with dextran-coated charcoal (DCC). 48 hrs later, ethanol or 1 nM R1881 was added to the cells. After an additional 48 hrs, the cells were subjected to proliferation assay, Western blotting, or immunocytochemistry.

### Peptide synthesis

All peptides used in this study were synthesized by ChiScientific, at ≥95% purity, and dissolved in 70% DMSO (ACROS Organic).

### Proliferation and apoptosis assays

For proliferation, cells were grown in medium containing 2% FBS extracted with dextran-coated charcoal (DCC). 48 hours later, ethanol or 1 nM R1881 was added to the cells followed by peptide treatment. The MTT assay (Sigma) was described as before [13]. For apoptosis, 5000 cells were seeded in 96-well plates and treated with Vehicle (DMSO) or Peptide B-8R for 8 hours at different concentrations. The Caspase (3/7) activity was measured using Apo-ONE Homogeneous Caspase-3/7 assay kit (Promega) and Caspase (3/7) cleavage by Western blotting. PARP cleavage was also used to measure cell apoptosis. Annexin V-FITC/Propidium Iodide (PI) double staining (Abcam) was also used to measure apoptosis. After treatment with Peptide B-8R, LNCaP cells were washed and stained for Annexin V/PI for 5 min in the dark and analyzed by LSRFortessa (BD Biosciences).

### Western blotting

Western blotting was performed as described using primary antibodies against sGCα1 (Cayman Chemical), pan-Akt (Cell Signaling Technology), phospho-AKT (S473, T308) (Cell Signaling Technology), Caspase3/7 (Santa Cruz Biotechnology), PARP (Cell Signaling Technology), p38 (Cell Signaling Technology), phosphor-p38 (T180/Y182, Cell Signaling Technology), β-Actin (Abcam).

### Immunocytochemistry

Immunocytochemistry was used to study the subcellular localization of sGCα1, Biotin-labeled Peptide B-8R in LNCaP cells. Anti-sGCα1 antibody (1:100 dilution; Santa Cruz Biotechnology), anti-Biotin antibody (1:200; Santa Cruz Biotechnology) were used for immunocytochemisty as described. Cells were seeded on glass coverslips, and washed in cold PBS, fixed by 3.7% Formaldehyde, and treated with 0.25% Triton-X100 for permeabilization. Primary antibody was incubated at 4° C for overnight. The next day, cells were washed with cold PBS and incubated with secondary antibody for 1.5 hours at 37° C, and then stained with DAPI for 5 minutes. At last, cells were mounted and observed under a fluorescence microscope (Olympus X81).

### Immunoprecipitation and Biotin pulldown

Immunoprecipitation (IP) in LNCaP cells was performed as described [15]. Whole-cell extracts from LNCaP cells were subjected to IP using Protein A/G plus Agarose (Santa Cruz). IP antibodies were against sGCα1 (Cayman Chemical), sGCβ1 (Cayman Chemical), or rabbit IgG (Santa Cruz) as a negative control. Biotin pulldown was performed as described before. For the competition assay, the same experiment was repeated with the following change: LNCaP cell extract was divided into 3 equal parts, with each part receiving 5 μg Peptide B-8R-Biotin and Vehicle, 150 μg Peptide C-8R, or 150 μg Peptide B-8R.

### ROS measurement and NAC treatment

Intracellular ROS levels were measured using the fluorescent probe 5-(and-6)- chloromethyl-2', 7'-dichlorodihydrofluorescein diacetate (CM-H2DCFDA) (Invitrogen). C81 cells were treated with Peptide B-8R at different concentration and ROS was measured as described before [18]. For NAC (N-acetyl cysteine) (Sigma) experiment, cells were pretreated with 5 mM NAC for 2 hours before adding Peptide B-8R or Peptide A-8R.

### cGMP ELISA assay

C81 cells were grown in the medium containing 5% FBS and treated with Peptide B-8R, C-8R or C-PTIO. At 24 hours of incubation, cell extract was boiled for 5 min in 0.05 M HCl (in PBS) and re-suspended in acetate buffer and cGMP level was measured by a cGMP ELISA kit (Biomedical Technologies) following the manufacturer’s protocol.

### Statistical analysis methods

All experiments were done at least three times and data are presented as averages plus standard deviations. The Student T-test was used to compare the difference between any pair of data. P < 0.05 was considered statistically significant.

## Results

### Peptide B is cytotoxic to prostate cancer cells that express sGCα1 and AR

**Fig 1:**
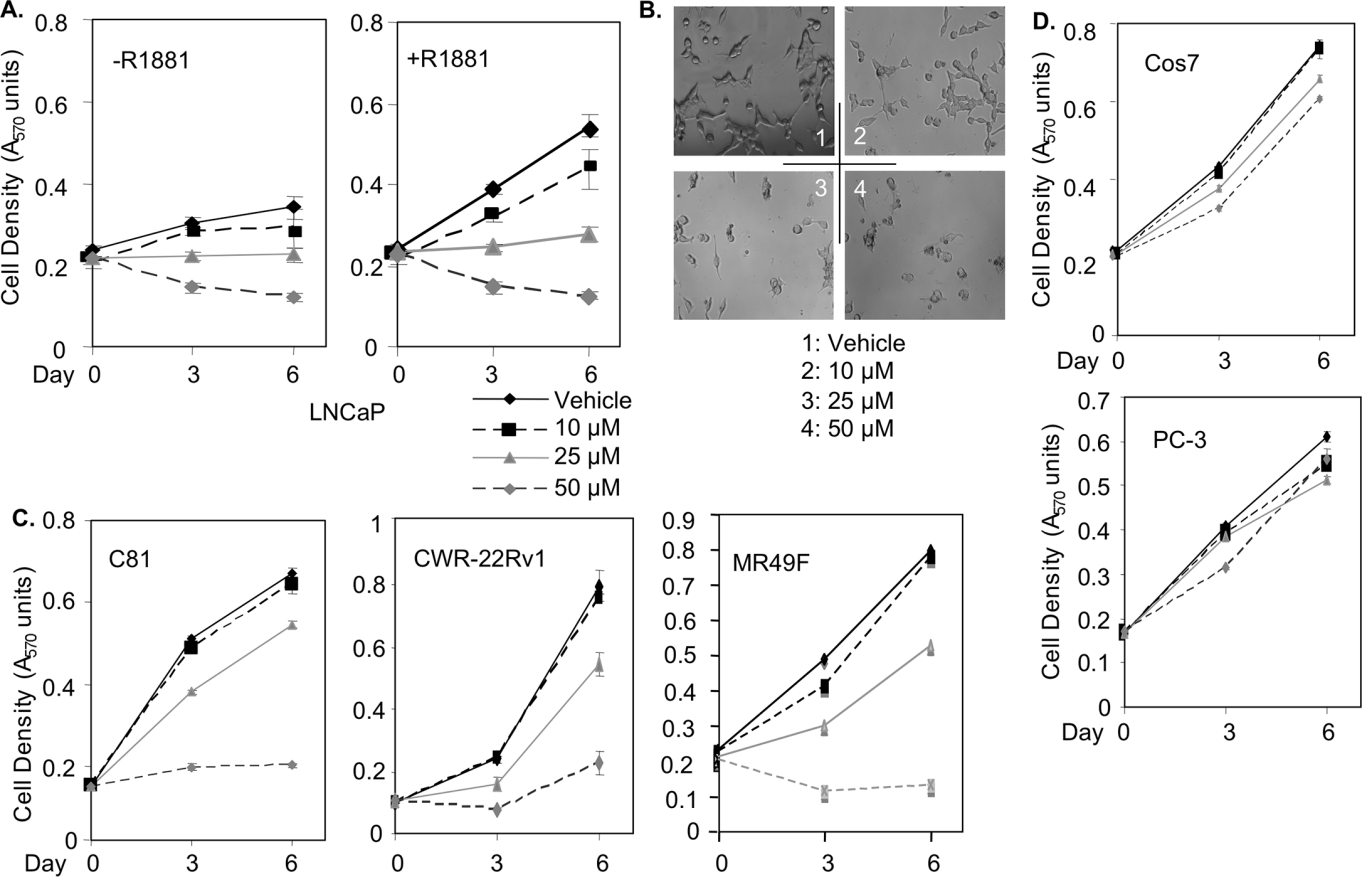
Peptide B-8R is strongly cytotoxic to prostate cancer cells. Vehicle or different concentrations of Peptide B-8R, as shown, was added to (A) hormone-dependent LNCaP cells grown in 2% serum without or with 1 nM R1881, (C) castration-resistant C81, CWR-22Rv1, or MR49F cells, or (D) sGCα1-deficient PC-3 or Cos cells. Cell number was measured after various days of incubation. Data points represent averages of three independent experiments plus standard deviations. Asterisks indicate statistical significance (P<0.03) of Peptide B-8R activity, relative to Vehicle. (B) LNCaP cells treated as described above were viewed by fluorescence microscopy.

As previously described [18], we designed several peptides to interfere with the pro-cancer functions of sGCα1. One of these peptides, Peptide B-8R, is described here to have strong cytotoxic activity. As shown in Fig 1A, Peptide B-8R blocked the proliferation of LNCaP cells in a dose-dependent manner; indeed, high concentrations of Peptide B-8R led to significant decreases in cell number (Fig 1A and 1B). While Peptide B-8R was strongly cytotoxic, Peptide B lacking the 8-Arginine sequence, a membrane translocation signal [19], was inactive (S1 Fig), showing that membrane translocation is necessary for Peptide B cytotoxicity. To study the peptide activity in hormone-independent, AR-positive prostate cancer cells, we used C81 [20] and CWR-22Rv1 cells [21]. Both hormone-independent cancer cell lines were sensitive to Peptide B-8R-induced cytotoxicity (Fig 1C), clearly demonstrating that Peptide B-8R can kill both hormone-dependent and -independent prostate cancer cells. Importantly, Peptide B-8R was also able to kill MR49F cells (Fig 1C), LNCaP cells that were selected for resistance to the second-generation Enzalutamide [22]. Further, Peptide B-8R is more potent in killing LNCaP cells than is Enzalutamide (S2A Fig), which together with the data on MR49F cells (see Fig 1C) strongly suggest that Peptide B-8R may be more effective at treating CRPC than Enzalutamide. Fig 1B illustrates that Peptide B-8R caused LNCaP cells to undergo a morphological change, becoming round and smaller and developing black spots in the cytoplasm. In contrast, PC-3 and Cos7 cells, which do not express sGCα1 [13], are weakly sensitive to Peptide B-8R (Fig 1D), clearly demonstrating that the peptide’s cytotoxicity is cell-specific and suggesting that it depends on sGCα1 expression.

### Peptide B induces apoptosis of prostate cancer cells

**Fig 2:**
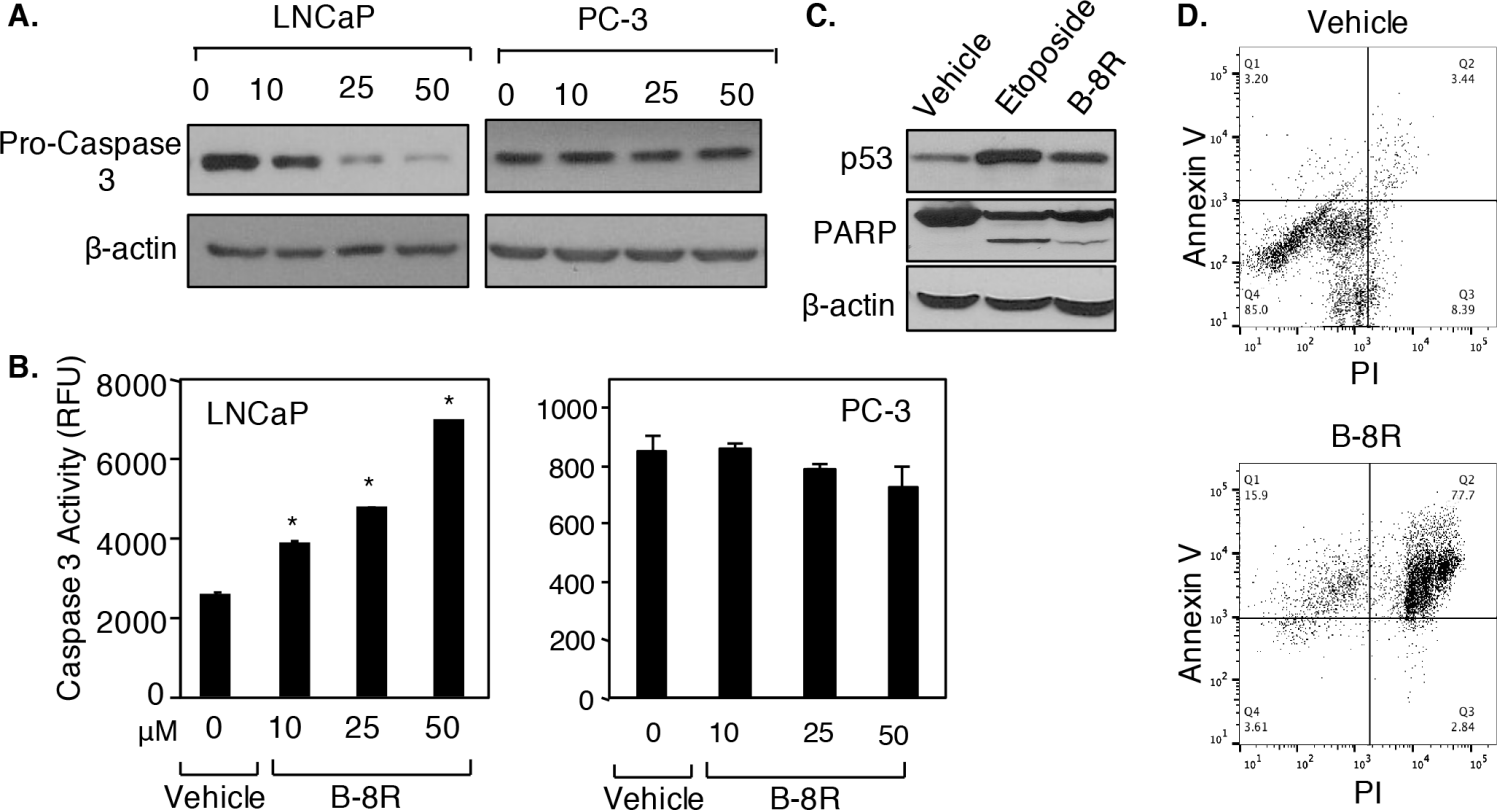
Peptide B-8R induces apoptosis and blocks tumorigenesis of prostate cancer cells. LNCaP or PC-3 cells were treated with Vehicle (0) or 10–25 μM Peptide B-8R, as shown, for 0–24 hrs and subjected to (A, B) Western blotting to measure either (A) Pro-Caspase 3 or (C) PARP cleavage or to (B) Caspase 3 assay, all of which monitored apoptosis. (D) LNCaP cells were treated with Vehicle or 50 μM Peptide B-8R for 1 hr and subjected to Annexin V/PI dual staining for apoptosis. Bar graphs represent averages of three independent experiments plus standard deviations. Asterisks indicate statistical significance (P<0.005) of Peptide B-8R activity, relative to Vehicle (0 μM). Note that β-actin was used to control for protein loading.

To determine mechanism of cytotoxicity, apoptosis was measured in Peptide B-8R-treated cells. As shown in Fig 2A, Peptide B-8R induced in a dose-dependent manner Pro-Caspase 3 cleavage in LNCaP cells but not PC-3 cells. Consistent with this, Peptide B-8R caused elevated Caspase 3 enzyme activity in LNCaP cells, but has no effect in PC-3 cells (Fig 2B). Peptide B-8R treatment also induced PARP cleavage and elevated p53 protein levels in LNCaP cells (Fig 2C), bolstering the Caspase 3 data and clearly demonstrating that Peptide B-8R induced apoptosis of LNCaP cells. Dual staining with Annexin V/PI provided a more quantitative measure of and stronger evidence for Peptide B-8R-induced apoptosis. As shown in Fig 2D, LNCaP cells treated with Vehicle are mainly found in Quadrant 1 (85%) having low Annexin V and PI staining, while treatment with Peptide A-8R led to most cells (77.7% in Quadrant 2) found in late apoptosis and 15.9% in early apoptosis (Quadrant 1). Thus, Peptide B-8R can induce within 1 hr the apoptosis of prostate cancer cells.

### Peptide B interacts with sGCα1 in prostate cancer cells

**Fig 3:**
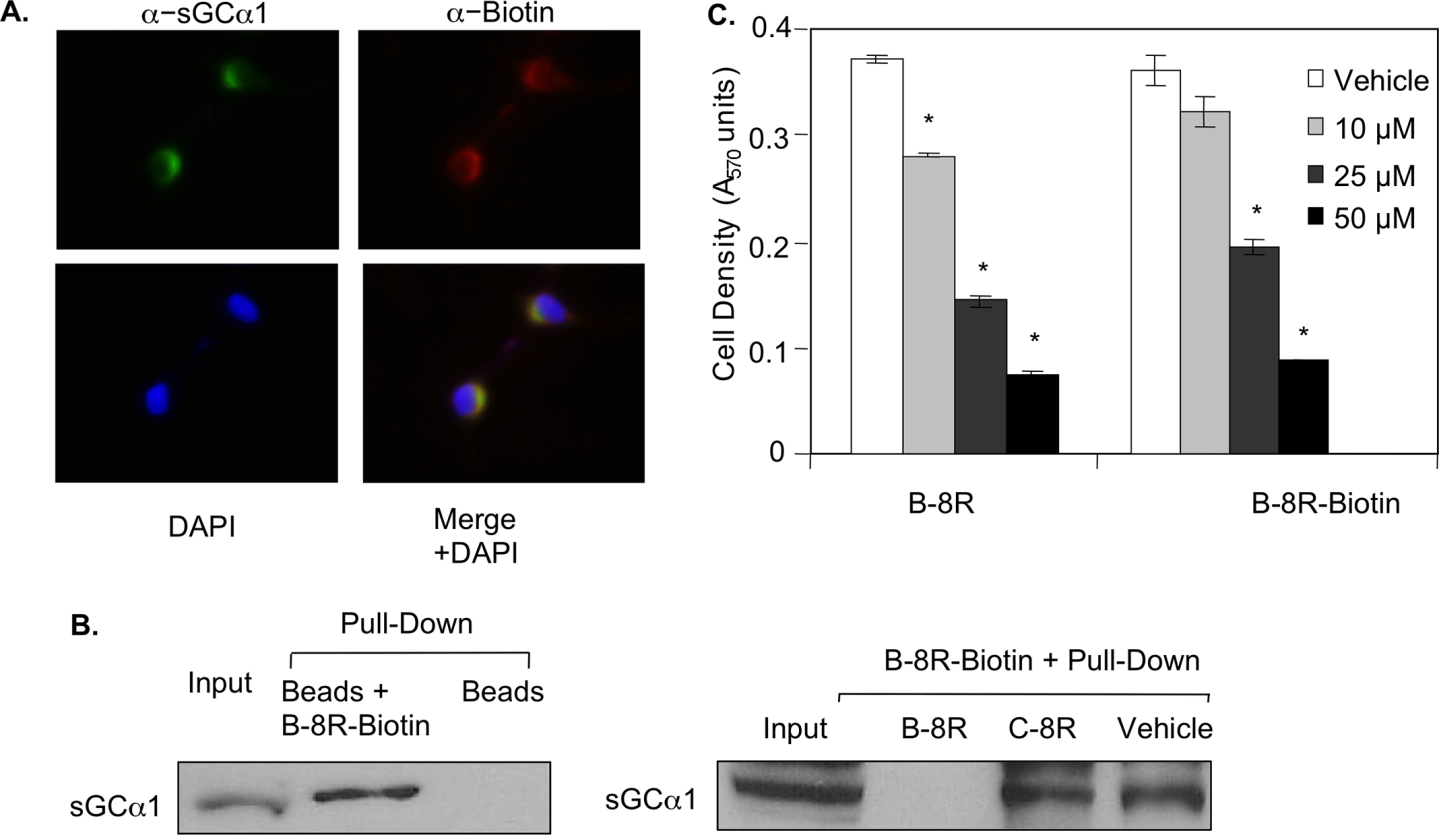
Peptide B-8R interacts with sGCα1 in prostate cancer cells. (A) LNCaP cells were treated with Biotin-labeled Peptide A-8R and subjected to immunocytochemistry using anti-sGCα1 or anti-Biotin antibody to measure subcellular co-localization of endogenous sGCα1 and Peptide B-8R. DAPI was used to stain nuclei. (B) LNCaP cytosolic extracts were incubated with Peptide B-8R-Biotin, plus or minus a 20-fold excess of unlabeled Peptide B-8R or C-8R, and subjected to purification using streptavidin-agarose. Western blotting was used to measure the co-purification of sGCα1. Asterisks indicate statistical significance (P<0.02) of Peptide B activity, relative to Vehicle.

Since Peptide B was designed to target sGCα1, it was expected to interact with sGCα1, which was studied using two complementary assays. First, LNCaP cells were treated with Biotin-tagged Peptide B-8R and subjected to immunocytochemistry using an antibody against Biotin and another against sGCα1. Consistent with what we observed before [13], sGCα1 was found exclusively in the cytoplasm of LNCaP cells. Biotin-tagged Peptide B was mainly present in the cytoplasm and co-localized with sGCα1 (Fig 3A), suggesting that Biotin-tagged Peptide B-8R interacts with endogenous sGCα1 in LNCaP cells. To confirm this interaction, in a second assay LNCaP cell extract was incubated with Biotin-tagged Peptide B-8R and subjected to Streptavidin-agarose pull-down, leading to co-purification of sGCα1 (Fig 3B). The specificity of this interaction was verified by repeating the pull-down experiment in which excess unlabeled Peptide B-8R or C-8R was added to the reaction with Biotin-tagged Peptide B-8R. Untagged Peptide B-8R was able to compete with Biotin-Peptide B for binding sGCα1, while untagged Peptide C-8R had no effect (Fig 3B). Addition of Biotin tag had no effect on the cytotoxic activity of Peptide B-8R (Fig 3C).

### Peptide B does not depend on nor affect NO signaling in prostate cancer cells

**Fig 4:**
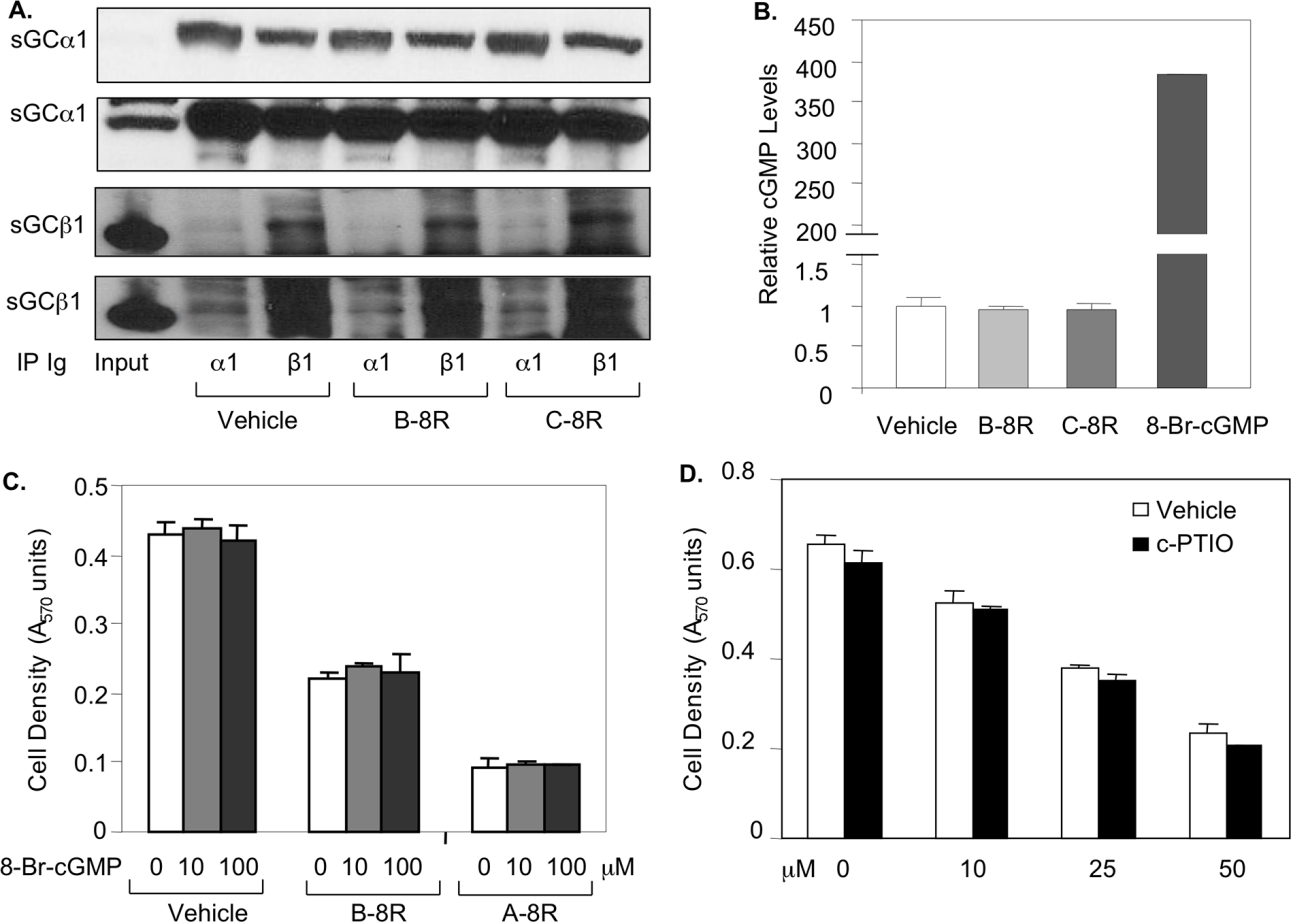
Peptide B-8R does not affect nor depend on NO signaling. (A) Whole-cell extracts from LNCaP cells treated with Vehicle, Peptide B-8R, or C-8R were subjected to IP using an anti-sGCα1 or anti-sGCα1 antibody and then probed by Western blotting for sGCα1 (Upper) or sGCβ1 (Lower). Input represents amount of sGCα1 or sGCβ1 found in extracts. Two different exposure times are shown for both sGCα1 and sGCβ1. (B) LNCaP cells grown in the absence or presence of 1 nM R1881 were treated with Vehicle, 20 μM Peptide B-8R, 20 μM C-8R, or 8-Br-cGMP and monitored for cGMP levels using the cGMP E1A kit (Enzo Life Sciences). (C) LNCaP cells grown in the absence or presence of 8-Br-cGMP were treated with Vehicle, Peptide B-8R, or A-8R and monitored for cell density using the MTT assay. (D) LNCaP cells were treated with Vehicle or different concentrations of Peptide A-8R and C-PTIO and monitored for cell density. Bar graphs represent averages of three independent experiments plus standard deviations. Asterisks indicate statistical significance (P<0.04) of cGMP levels of 8-Br-cGMP, relative to Vehicle.

Since the sGCα1 classic role is in NO signaling, we studied the possible involvement of this pathway in Peptide B-8R-induced cytotoxicity. First, we determined if Peptide A-8R affects NO signaling. As shown in Fig 4A, Peptide A-8R has no effect on sGCα1/sGCβ1 in vitro interaction, as measured using complementary immunoprecipitation (IP) experiments of sGCα1 or sGCβ1. Similarly, the cellular activity of the sGC enzyme, quantified by measuring cGMP levels, was not affected by Peptide B-8R (Fig 4B); 8-Br-cGMP was used to demonstrate the sensitivity of the cGMP assay. To determine if NO signaling affects Peptide A-8R cytotoxicity, we altered the levels of cGMP or NO. As shown in Fig 4C, treating cells with 8-Br-cGMP had no effect on cytotoxicity induced by Peptide B-8R or Peptide A-8R, which we have previously published [18]. Likewise, reducing NO levels by addition of c-PTIO did not affect Peptide B-8R cytotoxic activity (Fig 4D). Collectively, these data show that Peptide B-8R killing of prostate cancer cells neither affects nor depends on NO signaling.

### The cytotoxic activity of Peptide B does not depend on increased ROS, up-regulated p53, or phosphorylated p38 protein levels

**Fig 5:**
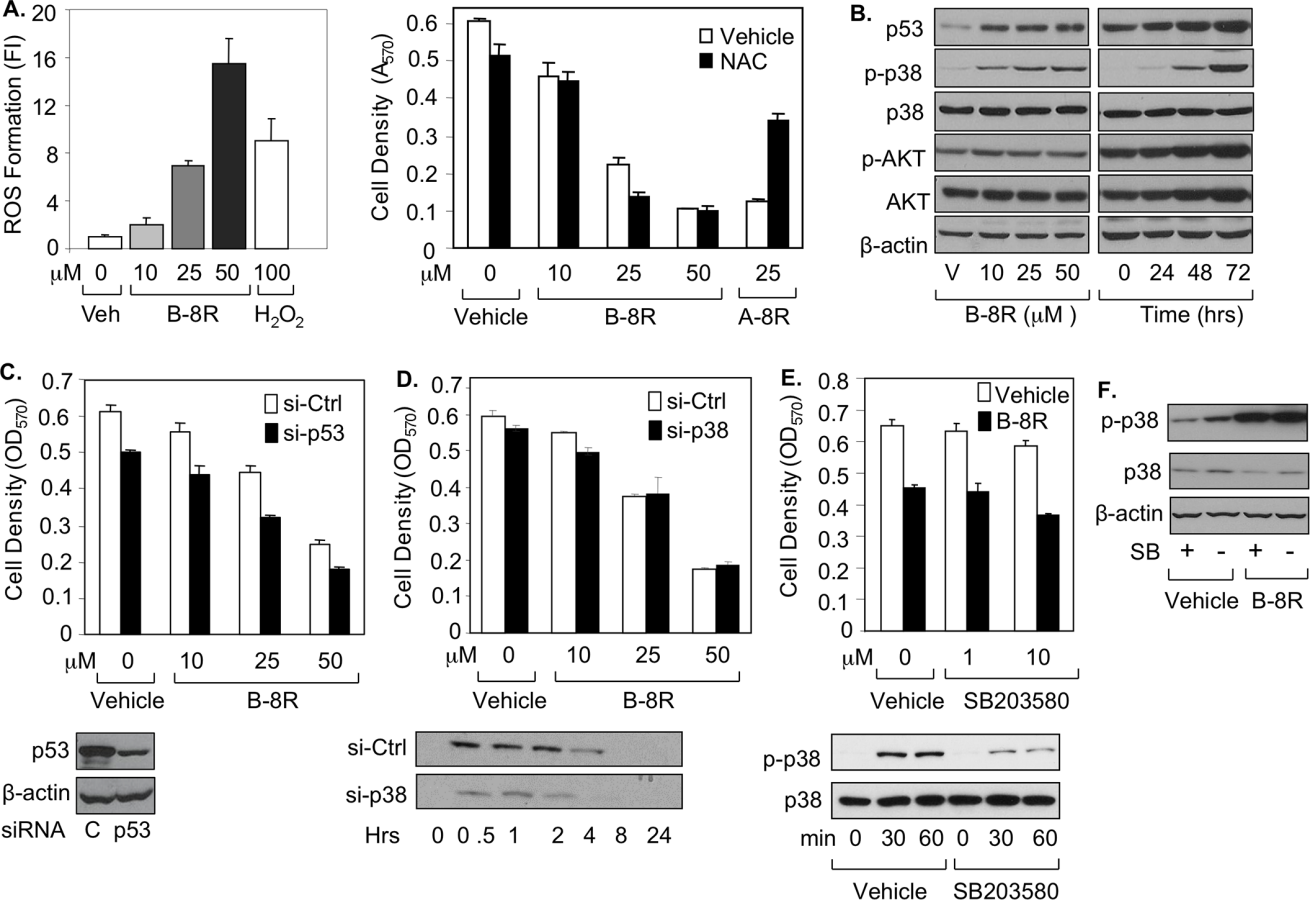
Peptide B-8R induces elevated ROS, p53, and activated p38 in prostate cancer cells. (A, B) C81 cells were treated with Vehicle, different μMolar concentrations of Peptide B-8R, or A-8R without or with 0–5 mM NAC (N-acetyl cysteine) and monitored for (A) ROS generation after 0–30 min as measured by fluorescence intensity (FI) or (B) cell number after 3 days of incubation as measured by MTT assay. (B) C81 cells were treated with Vehicle or different concentrations of Peptide B-8R, as shown, or 25 μM Peptide B-8R for 0–72 hrs and monitored for by Western blotting for expression of p53, P-p38, p38, p-AKT, or AKT. C81 cells were treated with different concentrations of Peptide B-8R, as shown, in the presence of (C) transfected p53 or Control (Ctrl) siRNA, (D) transfected p38 or Control (Ctrl) siRNA, or (E) Vehicle or 10 μM SB203580 and Western blotting is shown below each figure for p53, p-38, p-p38. Bar graphs represent averages of three independent experiments plus standard deviations. Asterisks indicate statistical significance (P<0.04) of (A) ROS Formation of Peptide B-8R or H_2_O_2_ relative to Vehicle or cell density of NAC-treated cells relative to Vehicle or (C) cell density of cells treated with si-p53 relative to si-Ctrl. (F) C81 cells were treated with Vehicle or 25 μM Peptide B-8R, in the absence or presence, as shown, 10 μM SB203580 (SB) and Western blotting was used to measure p-38 and p-p38. Note that β-actin was used to control for protein loading.

To determine the mechanism of Peptide B-8R cytotoxicity, we studied several signaling pathways that are activated by cytotoxic drugs. First, ROS generation was measured, and indeed Peptide B-8R was able to strongly induce ROS levels (Fig 5A), mimicking what was previously observed with Peptide A-8R [18]. The ROS-sequestering agent N-acetyl cysteine (NAC) (S3A Fig) was able to rescue prostate cancer cells treated with Peptide A-8R, as we have shown previously [18], but, interestingly, not Peptide B-8R (Fig 5A), clearly demonstrating that Peptide B-8R does not kill cancer cells by elevating ROS levels, as does Peptide A-8R [18]. These data also indicate that Peptides B-8R and A-8R kills cancer cells via different mechanisms, a contention supported by our finding that combined cytotoxic effect of both peptides together is greater than either peptide alone (S2B Fig).

Our earlier data (see Fig 2C) showed that Peptide B-8R induced elevated levels of p53, which we confirmed in Fig 5B and further demonstrated that the up-regulation is time-dependent. When p53 was depleted by siRNA in the context of Peptide B-8R treatment, there was no significant effect on the peptide’s cytotoxic activity (Fig 5C). Western blotting also showed that Peptide B-8R induced up-regulation of phosphorylated p38 (P-p38) protein, while there was no effect on AKT (Fig 5B). Once again, Peptide B-8R-treated cells were not rescued by either siRNA depletion of p38 (Fig 5D) or chemical inhibition of P-p38 by SB203580 (Fig 5E). Interestingly, the SB203580 inhibition of P-p38 was not observed when cells were treated with Peptide B-8R (Fig 5F), suggesting that Peptide B-8R-activated p38 is resistant to pharmacological inhibition by at least SB203580. As expected, 3-MA inhibition of PI3K, an activator of AKT, had no effect on Peptide B-8R activity (S3B Fig). Collectively, these data suggest that the elevated levels of p53 and P-p38 proteins are a consequence of rather than responsible for Peptide B-8R-induced cytotoxicity.

In view of the data above, we opted to study a series of chemicals to learn further about Peptide B-8R’s cytotoxic activity. To re-examine ROS, we used the free radical scavengers Trolox and Butylated Hydoxyanisole and the NADPH Oxidase inhibitor Diphenyleneiodonium Chloride, and, and just as with NAC (see Fig 5A), these anti-oxidants were unable to rescue Peptide B-8R-induced cytotoxicity and the latter two actually slightly cytotoxic to prostate cancer cells (S3 Fig), verifying that elevated ROS was not responsible the peptide’s cytotoxicity. Ferroptosis is a novel cell death pathway that depends on iron and ROS [23]. We found that inhibition of ferroptosis, using Ferrostatin and the iron chelators Hydroxyurea and Ciclopirox olamine, did not abolish the cytotoxicity induced by Peptide B-8R; indeed, Ciclopirox olamine was strongly cytotoxic on its own (S3 Fig). Similarly, inducing ferroptosis with Erastin or Ferric Citrate had no effect of the peptide’s cytotoxicity, with Erastin having a strong negative effect on the cells (S3 Fig). Thus, Peptide B-8R kills cells via a mechanism that does not depend on ferroptosis or elevated levels of p53 or P-p38.

## Discussion

Many novel AR-regulated genes have been discovered over the last few years, shedding light on how androgen-activated AR can have the multiple supportive functions in prostate cancer [1, 2]. Among the several genes our lab has discovered [12, 13, 24, 25], sGCα1 has been a focus because of its importance in the survival and growth of prostate cancer cells. Over-expression of exogenous sGCα1 in LNCaP cells drives their proliferation to a level that is observed with androgen treatment [13], demonstrating this one AR-regulated gene alone can mimic the growth effect of nearly 1000 genes that are regulated by androgen in LNCaP cells [13, 26]. One mechanism of sGCα1 action in prostate cancer cells is by associating with p53 and promoting its cytoplasmic sequestration, leading to cells that have enhanced survival and growth [15]. We more recently have determined that this complex of sGCα1 and p53 also contains CSN4 and CSN5 and CK2, and the enzyme activity of CK2 stabilizes the sGCα1 protein but destabilizes the p53 protein [16]. All these sGCα1 functions in prostate cancer cells are independent of NO signaling and sGCβ1, its dimerization partner in this signaling pathway [14].

The important pro-cancer functions of sGCα1 prompted us to design peptides to target this protein and disrupt these functions. We have already published data on one peptide, A-8R, which was determined to have potent cytotoxic and anti-tumor activity and kill prostate cancer cells via enhanced ROS generation [18]. Here we describe the activity of another peptide, B-8R, which is also cytotoxic to prostate cancer cells. Just like Peptide A-8R, Peptide B-8R does not affect nor depend on NO signaling. Although Peptide B-8R does indeed interact with sGCα1, it surprisingly does not disrupt sGCα1 interaction with sGCβ1. This is likely due to an sGCα1/sGCβ1 interaction that is mediated by multiple interaction domains on each protein and thus is of high affinity [27]. In addition, sGCα1 protein is over-expressed in both AR-expressing prostate cancer cells and prostate tumors, while sGCβ1 is weakly expressed [13], showing that there is a significant pool of endogenous sGCα1 that is free of sGCβ1 and therefore it is possible that Peptide B-8R targets this sGCα1 protein. Our published work has recently found that a large fraction of free sGCα1 is associated with p53, CSN4, CSN5, and CK2 [16], making it possible that Peptide B-8R may target this complex. Since this complex acts to destabilize p53 [16], Peptide B-8R would be expected to stabilize p53 if it disrupts the activity of this complex. Indeed, our data here clearly demonstrate that Peptide B-8R causes increased p53 protein levels in prostate cancer cells, consistent with the hypothesis that Peptide B-8R binds to sGCα1 and disrupts the complex activity on p53. Surprisingly, lowering p53 levels with siRNA does not rescue Peptide B-8R-treated cells, showing that elevated p53 is not responsible for the peptide’s cytotoxicity. Another effect of Peptide B-8R was to induce the phosphorylation and thus activation of p38, which was previously reported to induce apoptosis of prostate cancer cells [28]. It is interesting that our data show that Peptide B-8R activation of p38 happens even in the presence of the p38 chemical inhibitor SB203580, demonstrating that the peptide is able to abrogate the activity of this p38 inhibitor and perhaps others. Although this finding is interesting, it was not further studied because siRNA depletion of p38 failed to rescue Peptide B-8R-treated cells, showing that activation of p38 is not the mechanism by which the peptide’s kills cancer cells. While p53 up-regulation and p38 activation are unique effects of Peptide B-8R activity, elevated ROS generation is induced by both Peptide B-8R and A-8R [18]. Prostate cancer cells treated with Peptide A-8R-treated cells are strongly rescued by the ROS-sequestering agent NAC [18], but not at all when treated with Peptide B-8R, demonstrating that Peptide B-8R elevated ROS generation is not responsible for its cytotoxicity. Collectively, our data here show that Peptide B-8R induces p53 up-regulation, p38 activation, and ROS generation, but none of these effects is the cause of the peptide cytotoxic activity on prostate cancer cells. Our study further shows that ferroptosis, a cell death pathway that depends on iron and ROS [23], is also not involved in the cytotoxicity of Peptide B-8R. Further studies are required to determine the mechanism of cytotoxic action of Peptide B-8R.

It is significant that Peptide B-8R kills prostate cancer cells that express AR and sGCα1, since these cancer cells represent the large majority of prostate tumors [13, 29]. We show that both androgen-dependent and–independent prostate cancer cells are sensitive to Peptide B-8R toxicity. This includes the androgen-independent MR49F cells, which are resistant to the promising new prostate cancer drug Enzalutamide [22], and our finding that these cells are sensitive to Peptide B-8R suggests that Peptide B-8R may be effective against CRPC, the lethal form of prostate cancer. These activities warrant further investigation in the future into Peptide B-8R-induced cytotoxicity. This work can lead to further modifications of Peptide B-8R to make it more potent and/or stable or lead to small molecule analogs that are more feasible to use clinically.

## Supporting information

S1 Fig Peptide B-8R cytotoxicity depends on membrane translocation. LNCaP cells were treated with Vehicle or different concentrations of Peptide B-8R or Peptide A and monitored for cell density using the MTT assay. Bar graphs represent averages of three independent experiments plus standard deviations. Asterisks indicate statistical significance (P<0.005).
(TIF)

S2 Fig Peptides B-8R and A-8R additively kill prostate cancer cells. (A) LNCaP cells were treated with Vehicle or different concentrations of Peptide B-8R or Enzalutamide, as shown, or (B) C81 or MR49F cells treated with Vehicle or different concentrations of Peptide B-8R and/or A-8R, as shown, and monitored for cell density using the MTT assay. Bar graphs represent averages of three independent experiments plus standard deviations. Asterisks indicate statistical significance (P<0.005).
(TIF)

S3 Fig Peptides B-8R cytotoxicity of prostate cancer cells does not depend on ferroptosis. (A) LNCaP cells were treated with Vehicle or 25 μM of Peptide B-8R in the absence (Vehicle) or presence of 100 μM Trolox (Tro), 100 μM Butylated Hydoxyanisole (Bha), 0.5 μM Diphenyleneiodonium Chloride, (DIC), Hydroxyurea (Hu) 5 μM Ciclopirox olamine (Cpo), 25 μM Ferrostatin (Fer1), 10 μM Erastin, 25 μM Ferric citrate (Fc), or 5 mM 3-Methyladenine (3-MA), as shown, and monitored for cell density using the MTT assay. Bar graphs represent averages of three independent experiments plus standard deviations. Asterisks indicate statistical significance (P<0.005).
(TIF)
