# Supplementary figures and images for: Peptide B targets soluble guanylyl cyclase α1 and kills prostate cancer cells
Source: PLoS One. 2017 Aug 31;12(8):e0184088. doi: 10.1371/journal.pone.0184088 (PMC5578680; doi:10.1371/journal.pone.0184088)

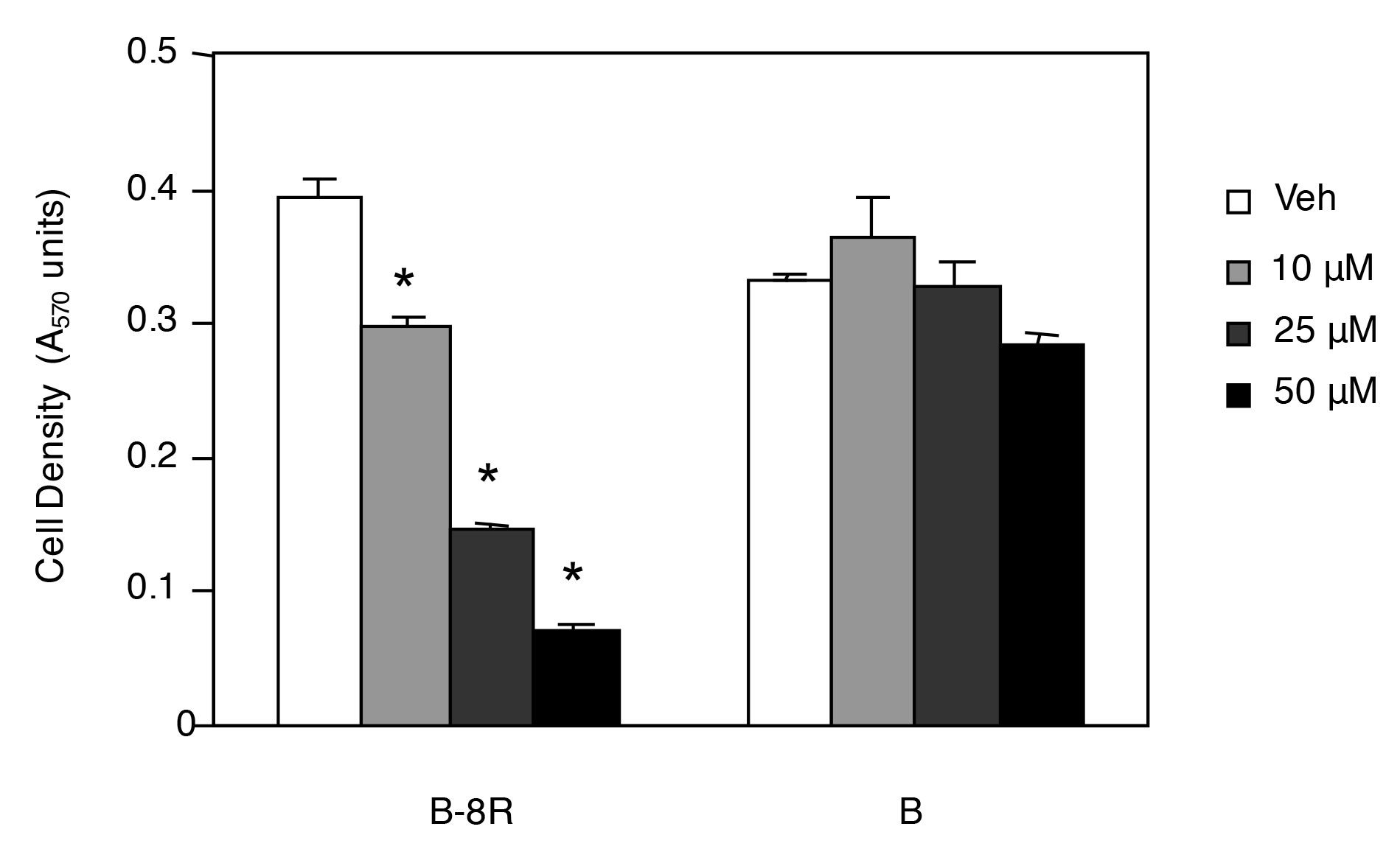

Supplement: S1 Fig — LNCaP cells were treated with Vehicle or different concentrations of Peptide B-8R or Peptide A and monitored for cell density using the MTT assay. Bar graphs represent averages of three independent experiments plus standard deviations. Asterisks indicate statistical significance (P<0.005). (TIF) [file pone.0184088.s001.tif]

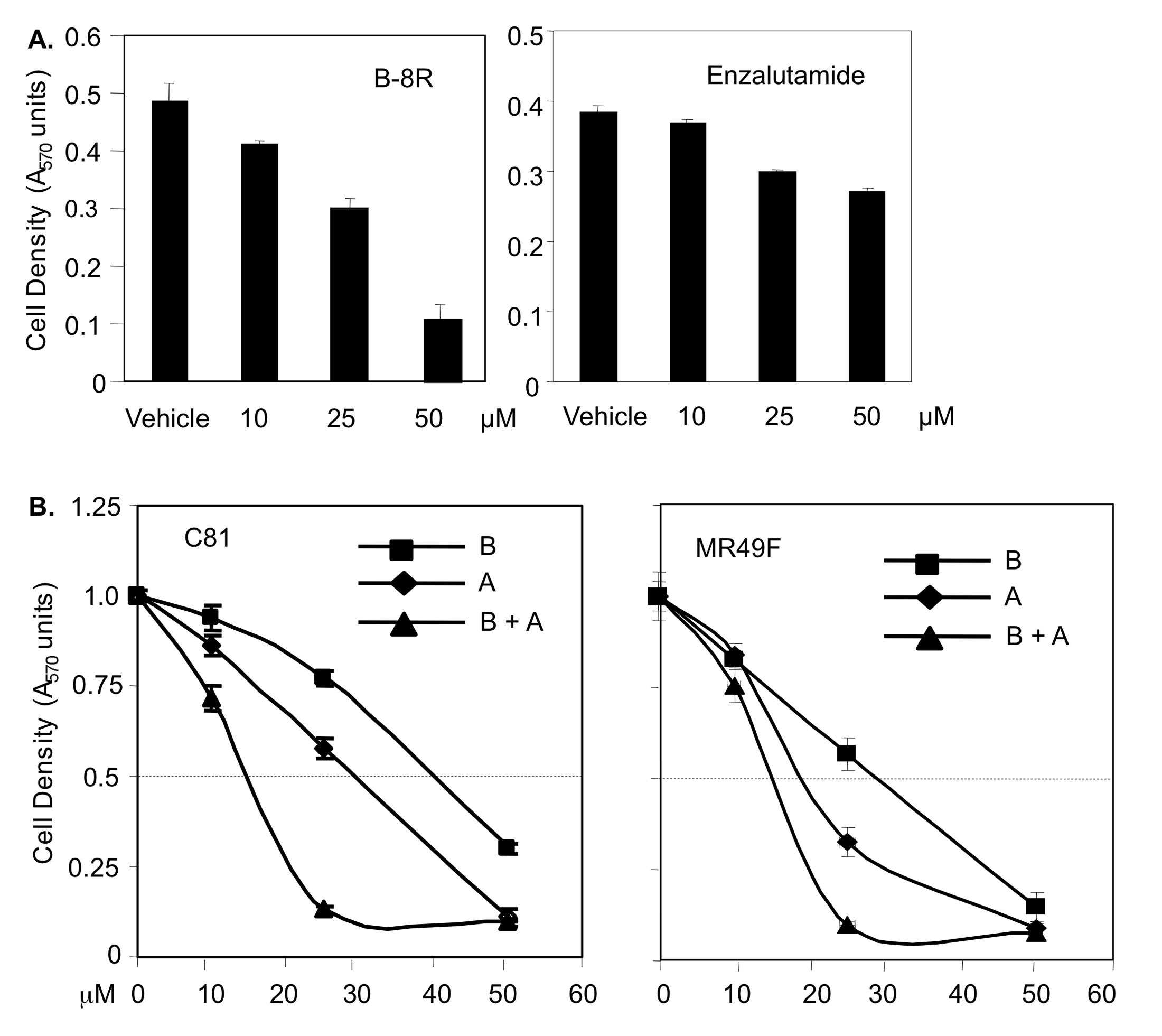

Supplement: S2 Fig — (A) LNCaP cells were treated with Vehicle or different concentrations of Peptide B-8R or Enzalutamide, as shown, or (B) C81 or MR49F cells treated with Vehicle or different concentrations of Peptide B-8R and/or A-8R, as shown, and monitored for cell density using the MTT assay. Bar graphs represent averages of three independent experiments plus standard deviations. Asterisks indicate statistical significance (P<0.005). (TIF) [file pone.0184088.s002.tif]

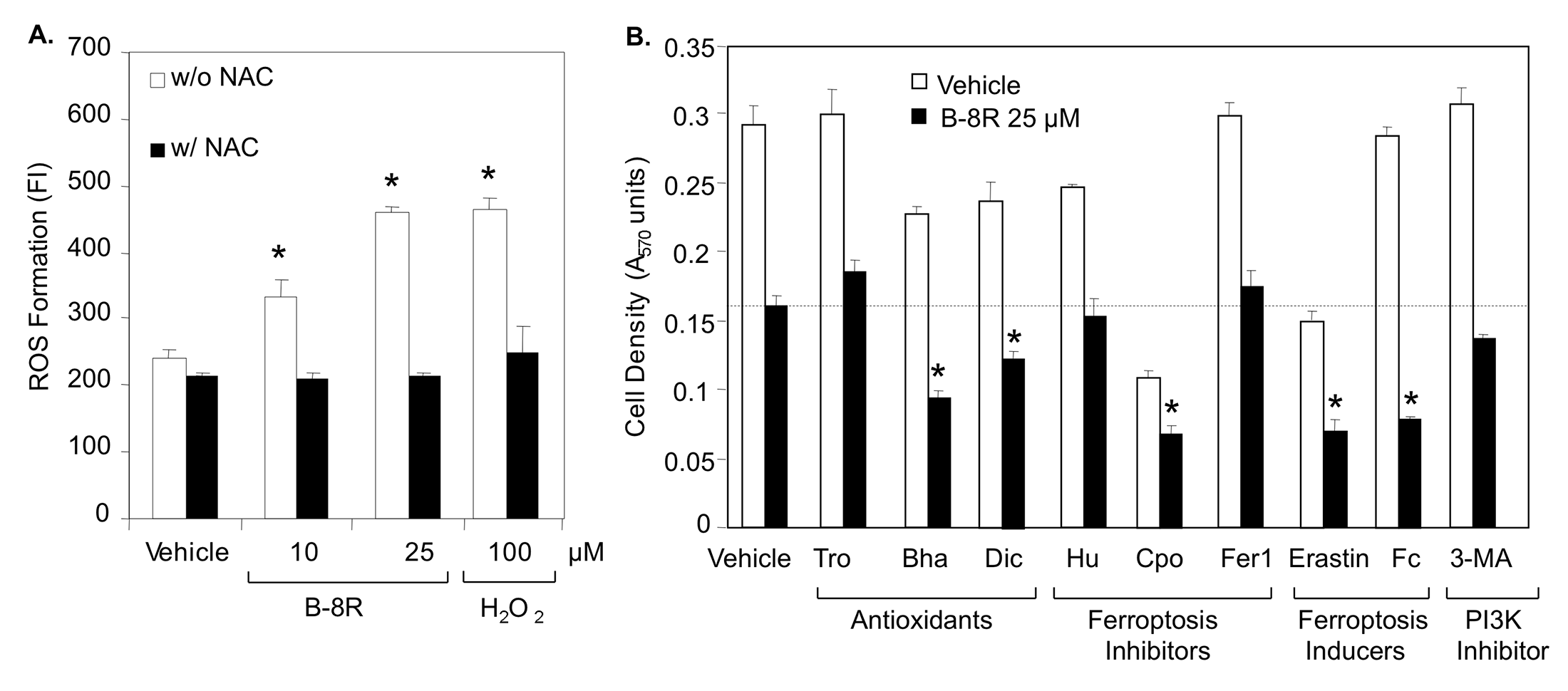

Supplement: S3 Fig — (A) LNCaP cells were treated with Vehicle or 25 μM of Peptide B-8R in the absence (Vehicle) or presence of 100 μM Trolox (Tro), 100 μM Butylated Hydoxyanisole (Bha), 0.5 μM Diphenyleneiodonium Chloride, (DIC), Hydroxyurea (Hu) 5 μM Ciclopirox olamine (Cpo), 25 μM Ferrostatin (Fer1), 10 μM Erastin, 25 μM Ferric citrate (Fc), or 5 mM 3-Methyladenine (3-MA), as shown, and monitored for cell density using the MTT assay. Bar graphs represent averages of three independent experiments plus standard deviations. Asterisks indicate statistical significance (P<0.005). (TIF) [file pone.0184088.s003.tif]
